# Fear of hypoglycemia and associated factors in hospitalized patients with type 2 diabetes: a cross‑sectional study

**DOI:** 10.1038/s41598-022-24822-1

**Published:** 2022-11-25

**Authors:** Jing Huang, Wei Peng, Shenglan Ding, Shuyuan Xiong, Zhiping Liu

**Affiliations:** 1 Department of Endocrinology, The First Affiliated Hospital of Chongqing Medical University, 1st YouYi Rd, Yuzhong District, Chongqing, 400016 China; 2 Department of Nursing, Chengdu Women’s and Children’s Central Hospital, School of Medicine, University of Electronic Science and Technology of China, Chengdu, 610091 China; 3 School of Nursing, Peking University, Beijing, China

**Keywords:** Endocrinology, Public health

## Abstract

The present cross-sectional survey was performed to assess the prevalence and factors associated with fear of hypoglycemia (FoH) in hospitalized patients with type 2 diabetes (T2D). Between July and December 2020, 494 patients with T2D were evaluated via structured questionnaires containing sociodemographic information, clinical information, and the Fear of Hypoglycemia-15 scale (FH-15). Patients were divided into the FoH and non-FoH groups according to the FH-15 score. Univariate and multivariate logistic regression analyses were performed to determine factors associated with FoH. Variables with *P* values < 0.1 in the univariate model were included in the multivariate model. In this study, the prevalence of FoH was 17.4% (86/494). 247 (50.0%) patients experienced hypoglycemic episodes in the past year, and 15 (3.0%) patients experienced severe hypoglycemic episodes in the past year. The mean age was 60.04 ± 11.71 years old, and female patients accounted for 39.9% of the sample. The item with the highest average FH-15 scores was: how often are you afraid of having hypoglycemia while alone? Multivariate logistic regression analysis indicated that living alone (OR 2.48; 95% CI 1.20–5.14; *P* = 0.015), number of hypoglycemic episodes in the past year (OR 1.06; 95% CI 1.03–1.10; *P* < 0.001), number of severe hypoglycemic episodes in the past year (OR 2.61; 95% CI 1.20–5.69; *P* = 0.016), and duration of insulin use (OR 1.06; 95% CI 1.02–1.10; *P* = 0.006) were associated with FoH. The prevalence of FoH in hospitalized patients with T2D was high. FoH was associated with living alone, number of hypoglycemic episodes in the past year, number of severe hypoglycemic episodes in the past year, and duration of insulin use. These findings can contribute to early decision-making for preventing, identifying, and improving FoH in patients with T2D. In the future, interventions aimed at reducing FoH to improve the harmful effects of FoH are necessary, such as increasing diabetes-related knowledge and skills, increasing social support, reducing psychological fear, and minimizing risks for hypoglycemic episodes.

## Introduction

China currently has the most significant number of patients with diabetes worldwide, and the number of patients with diabetes is continuing to rise^1^. The goal of diabetes treatment and care is to improve the metabolic control of patients and prevent and slow the occurrence and development of microvascular and macrovascular complications^2,3^. To achieve this goal, patients with diabetes will consider changing their lifestyles and receiving insulin or hypoglycemic drugs. However, the treatment measures required to control blood glucose at the ideal level result in increased risks for hypoglycemia, particularly in patients who use insulin or insulin secretagogues^4,5^.

Hypoglycemia can lead to unpleasant symptoms, such as tremulousness, sweating, palpitations, hunger, cognitive dysfunction, behavioral changes, seizures, and loss of consciousness^6,7^. Moreover, patients who develop hypoglycemia are at greater risk of cardiovascular events and death^8^. The apparent discomfort, personal harm, and unpredictability of hypoglycemia may cause patients to fear hypoglycemia^9^. Fear of hypoglycemia (FoH) worries patients with diabetes about hypoglycemia and its negative consequences, such as the harm caused by hypoglycemia and the adverse effects of changes in cognition, behavior, and consciousness^10^. Patients with FoH are likely to engage in avoidance behavior to prevent hypoglycemia^11^, for instance, reducing the insulin dosage, increasing food intake, and restricting activities, which may harm their glycemic control^11,12^. In addition, FoH was positively correlated with negative emotions such as psychological distress and anxiety in patients with diabetes^13,14^ and reduced the quality of life^15,16^. Therefore, FoH is a critical psychological obstacle to metabolic control, negatively influencing the self-management and mental health of patients with diabetes.

Currently, there are many studies on FoH in patients with type 1 diabetes (T1D), probably because that T1D, as insulin-dependent diabetes, has a higher incidence of hypoglycemia than type 2 diabetes (T2D)^17,18^. However, due to the high prevalence of T2D, the popularization of intensive glycemic control, and the increasing prominence of psychological problems in patients with T2D^3,18–20^, research related to FoH in patients with T2D has gradually increased^21–23^. At present, the exploration of FoH in China is still in its infancy, which limits the further development of targeted intervention strategies for FoH. Therefore, our purpose was to assess the prevalence of FoH in hospitalized patients with T2D and identify potential factors associated with FoH to provide a theoretical foundation for health care providers to prevent, identify, and reduce FoH in patients with T2D.

## Methods

### Ethics approval and informed consent

This study was approved by the ethics committee of the First Affiliated Hospital of Chongqing Medical University (ID: 2020-418), and the procedure was conducted following the Declaration of Helsinki. Each patient in the study read and signed a written informed consent form before data collection.

### Study design and participants

This cross-sectional study was carried out at the First Affiliated Hospital of Chongqing Medical University in Chongqing Municipality, a city in Southwest China, from July to December 2020. Hospitalized patients in the Department of Endocrinology who met the following criteria were recruited via convenience sampling. The inclusion criteria were as follows: (1) T2D, diagnosed via the criteria of WHO in 1999; (2) age ≥ 18 years old; (3) diabetes duration ≥ one year; (4) able to provide informed consent and communicate with researcher; and (5) willing to participate in this study. Subjects who were pregnant, had a history of mental illness, or were too sick to complete the investigation (such as dyspnea, palpitation, paralysis, headache, dizziness, blurred vision) were excluded. The history of mental illness was determined from the patient's medical records and verified with the patients and their families face-to-face or by phone. The main reasons for hospitalization of these patients with T2D were hyperglycemia and chronic complications, and a small number of patients were hospitalized for acute complications, infections, and hypoglycemia. The sample size was estimated using a rule of thumb for sample size calculation in logistic regression analysis, i.e., ten times the number of variables^24^. There were 40 variables in the study. Considering the 10% invalid questionnaire rate, a sample size ≥ 444 was appropriate.

### Data collection

Before the start of the survey, four researchers had received uniform training. Questionnaires were distributed and collected on the spot to ensure the validity and authenticity of the data. First, the researchers explained the goal and methods of the survey to eligible patients. Second, the patients willing to participate in this study needed to understand and sign a written informed consent. Third, the researchers used standard instructions to guide the patients in filling out the questionnaires, and the researchers provided help when the patients had difficulty filling out the questionnaires. After that, the researchers checked whether the questionnaires were completed item by item. If it was not completed, the researchers returned it to the patients and reminded them to check it carefully. Finally, the researchers collected the questionnaires and saved them after numbering.

### Assessment of sociodemographic and clinical variables

The sociodemographic variables considered in this study included age, sex, marital status, sources of medical expenses, residential location, educational level, job status, monthly household income per capita, annual self-funded medical expenses, cohabitation status, and hypoglycemia health education. The clinical variables considered in this study included body mass index (BMI), glycated hemoglobin (HbA1c), blood pressure, treatment in the past year, duration of insulin use, duration of diabetes, family history of diabetes, type and number of hypoglycemic drugs used in the past year, type and number of diabetes complications, hypoglycemic episodes, and impaired awareness of hypoglycemia (IAH). A plasma glucose level ≤ 3.9 mmol/L was regarded as hypoglycemia in patients with diabetes. Severe hypoglycemia was defined as any hypoglycemic episode that led to seizures, loss of consciousness, or urgent medical attention^25^. Hypoglycemic episodes, such as number of hypoglycemic episodes in the past two weeks, number of hypoglycemic episodes in the past year, number of severe hypoglycemic episodes in the past year, and number of severe hypoglycemic episodes since diagnosis, were self-reported by patients on a questionnaire based on blood glucose self-monitoring diaries and their memories.

BMI was calculated by dividing weight (kg) by height (m) squared. Blood pressure was recorded in the sitting position after 5 min of rest by the validated medical electronic blood pressure monitor (Omron; HBP-1300)^26^. Blood samples were collected after fasting for at least 8 h. The HbA1c levels were determined by high-performance liquid chromatography. Diabetic nephropathy included a urinary albumin/creatinine ratio (UACR) ≥ 30 mg/g or an estimated glomerular filtration rate (eGFR) < 90 ml·min^−1^·(1.73 m^2^)^−1^. Diabetic retinopathy included any grade of diabetic retinopathy or maculopathy during the fundus examination. Diabetic peripheral neuropathy was diagnosed by the neuropathy symptom score (NSS) and neuropathy disability score (NDS). Diabetic foot included foot infections, ulcers, or deep tissue destruction. Diabetic macroangiopathy included atherosclerosis and arterial stenosis of the blood vessels of the heart, neck, or lower extremities.

### Assessment of FoH in patients with T2D

FoH was evaluated using the Fear of Hypoglycemia-15 scale (FH-15)^27^, validated in the Chinese population^28^. Patients in this study answered the FH-15 on a self-administered form. The FH-15 is a 15-item instrument, and each item is scored from 1 (never) to 5 (every day), comprising three factors: fear, interference, and avoidance. The cutoff score of FoH is 30.5 in the Chinese version of the FH-15^28^. Cronbach’s alpha was 0.949 in our sample.

### Assessment of IAH in patients with T2D

Hypoglycemia awareness was evaluated according to the Gold score, a visual analog scale ranging from 1 to 7^29,30^. A Gold score ≥ 4 indicates IAH.

### Statistical analysis

The FH-15 scores were categorized into dichotomous groups according to the cutoff score of FoH. Descriptive statistics of the sociodemographic and clinical variables were performed. The mean (standard deviation, SD) was used to express the continuous variables. The independent sample t-test for normally distributed variables and the Mann–Whitney *U* test for nonnormally distributed variables were performed to analyze group differences. Frequencies and percentages were used to express the categorical variables. Pearson's chi-squared test, the continuity corrected chi-squared test, or Fisher’s exact test was performed to analyze group differences. Post hoc analyses were conducted using Bonferroni correction to examine the group differences in each category. Logistic regression analysis was used to explore the factors associated with FoH. In univariate logistic regression analysis, variables with *P* values < 0.1 were included in multivariate logistic regression analysis (forward LR method). Epidata software (version 3.1) was used for data entry. All statistical analyses were carried out in SPSS software (version 23.0). A two-sided *P* value < 0.05 was considered statistically significant.

## Results

### Sociodemographic characteristics of patients with T2D

A total of 515 patients with T2D were selected and invited to participate in this survey, and 21 questionnaires were invalid. Thus, 494 patients were enrolled in the data analysis. The sociodemographic characteristics of the participants are listed in Table 1. The mean age of the patients was 60.04 ± 11.71 years old. Most patients were male (n = 297; 60.1%), married (n = 428; 86.6%), living with others (n = 452, 91.5%), and living in urban areas (n = 462; 93.5%). 458 (92.7%) patients had health insurance, and 310 (62.8%) patients had received hypoglycemia health education. In addition, the proportion of female patients and patients living alone in the FoH group was higher than that in the non-FoH group (*P* = 0.035 and *P* = 0.004). Annual self-funded medical expenses were significantly associated with FoH (*P* = 0.028). Post hoc analysis found that the prevalence of FoH in the group with annual self-funded medical expenses > 5000 China Yuan (CNY) was higher than that in the group with annual self-funded medical expenses < 2000 CNY (*P* = 0.011; Bonferroni correction *P* = 0.0167). Other sociodemographic characteristics did not differ significantly between the FoH and non-FoH groups.

**Table 1:** Sociodemographic characteristics of patients with type 2 diabetes.

Variable	Total (n = 494)	FoH (n = 86)	non-FoH (n = 408)	*P* value
Age (Mean ± SD, years)	60.04 ± 11.71	61.30 ± 11.20	59.77 ± 11.81	0.270
**Sex, n (%)**				0.035
Male	297 (60.1)	43 (50.0)	254 (62.3)	
Female	197 (39.9)	43 (50.0)	154 (37.7)	
**Marital status, n (%)**				0.057
Single	11 (2.2)	3 (3.5)	8 (2.0)	
Married	428 (86.6)	70 (81.4)	358 (87.7)	
Divorced	16 (3.2)	1 (1.2)	15 (3.7)	
Widowed	39 (7.9)	12 (14.0)	27 (6.6)	
**Residential location, n (%)**				0.242
Urban	462 (93.5)	78 (90.7)	384 (94.1)	
Rural	32 (6.5)	8 (9.3)	24 (5.9)	
**Educational level, n (%)**				0.144
Elementary school	67 (13.6)	15 (17.4)	52 (12.7)	
Middle school	132 (26.7)	26 (30.2)	106 (26.0)	
High school	120 (24.3)	12 (14.0)	108 (26.5)	
Junior college	82 (16.6)	17 (19.8)	65 (15.9)	
Undergraduate college	93 (18.8)	16 (18.6)	77 (18.9)	
**Job status, n (%)**				0.210
Full-time	153 (31.0)	21 (24.4)	132 (32.4)	
Part-time	4 (0.8)	1 (1.2)	3 (0.7)	
Retired	312 (63.2)	57 (66.3)	255 (62.5)	
Other (job-waiting, etc.)	25 (5.1)	7 (8.1)	18 (4.4)	
**Monthly household income per capita, n (%)**				0.122
< 1000 CNY (< 158.5 USD)	15 (3.0)	5 (5.8)	10 (2.5)	
1000–2999 CNY (158.5–475.4 USD)	128 (25.9)	28 (32.6)	100 (24.5)	
3000–5000 CNY (475.5–792.5 USD)	192 (38.9)	30 (34.9)	162 (39.7)	
> 5000 CNY (> 792.5 USD)	159 (32.2)	23 (26.7)	136 (33.3)	
**Annual self-funded medical expenses, n (%)**				0.028
< 2000 CNY (< 317.0 USD)	80 (16.2)	7 (8.1)	73 (17.9)	
2000–5000 CNY (317.0–792.5 USD)	171 (34.6)	27 (31.4)	144 (35.3)	
> 5000 CNY (> 792.5 USD)	243 (49.2)	52 (60.5)	191 (46.8)	
**Sources of medical expenses**				0.811
Self-funded	10 (2.0)	2 (2.3)	8 (2.0)	
Medical insurance	458 (92.7)	79 (91.9)	379 (92.9)	
New rural cooperative medical system	26 (5.3)	5 (5.8)	21 (5.1)	
**Cohabitation status, n (%)**				0.004
Living with others	452 (91.5)	72 (83.7)	380 (93.1)	
Living alone	42 (8.5)	14 (16.3)	28 (6.9)	
Hypoglycemia health education, n (%)	310 (62.8)	60 (69.8)	250 (61.3)	0.139

FoH, fear of hypoglycemia; CNY, China Yuan; USD, US dollars; SD, standard deviation. The independent sample t-test, Pearson’s chi-squared test, and Fisher’s exact test were used to compare differences between groups.

### Clinical characteristics of patients with T2D

Table 2 represents the clinical characteristics of the participants. The average BMI, HbA1c, systolic and diastolic blood pressure of the patients were 24.30 ± 3.31 kg/m^2^, 7.91 ± 2.03%, 132.98 ± 16.36 mmHg, and 77.59 ± 10.13 mmHg, respectively. Approximately half of the patients had a family history of diabetes (n = 260; 52.6%). Compared with the group without FoH, the group with FoH had more hypoglycemic episodes in the past two weeks (*P* < 0.001) and the past year (*P* < 0.001) and had more severe hypoglycemic episodes in the past year (*P* = 0.002). Diabetes treatment in the past year had a significant correlation with FoH (*P* = 0.034). Post hoc multiple comparisons showed that the prevalence of FoH in the hypoglycemic drugs combined with insulin therapy group was higher than that in the hypoglycemic drugs group (*P* = 0.007; Bonferroni correction *P* = 0.0083). Further, the prevalence of FoH was higher in patients using insulin or insulin secretagogues than in patients using other glycemic control measures. Compared with the group without FoH, the group with FoH had a longer duration of insulin use (*P* < 0.001), longer duration of diabetes (*P* = 0.029), more diabetes complications (*P* = 0.005), and a larger proportion of diabetic retinopathy (*P* = 0.032) and diabetic peripheral neuropathy (*P* = 0.002). Among the patients included in the analysis, 332 patients had a history of hypoglycemia and completed the assessment of the Gold score^29^. According to the Gold score, 65 (13.2%) patients had IAH. The proportion of IAH in the FoH group was significantly higher than that in the non-FoH group (*P* = 0.046). Other clinical characteristics did not differ significantly between the FoH and non-FoH groups.

**Table 2:** Clinical characteristics of patients with type 2 diabetes.

Variable	Total (n = 494)	FoH (n = 86)	non-FoH (n = 408)	*P* value
BMI (Mean ± SD, kg/m^2^)	24.30 ± 3.31	24.54 ± 2.99	24.25 ± 3.38	0.457
HbA1c (Mean ± SD, %)	7.91 ± 2.03	7.76 ± 1.95	7.95 ± 2.05	0.435
Systolic blood pressure (Mean ± SD, mmHg)	132.98 ± 16.36	135.38 ± 16.25	132.47 ± 16.36	0.134
Diastolic blood pressure (Mean ± SD, mmHg)	77.59 ± 10.13	76.90 ± 10.10	77.73 ± 10.14	0.488
Number of hypoglycemic episodes in the past two weeks (Mean ± SD)	0.40 ± 1.17	0.92 ± 1.46	0.29 ± 1.07	< 0.001
Number of hypoglycemic episodes in the past year (Mean ± SD)	2.87 ± 6.70	6.69 ± 10.80	2.07 ± 5.13	< 0.001
Number of severe hypoglycemic episodes in the past year (Mean ± SD)	0.04 ± 0.25	0.12 ± 0.45	0.02 ± 0.18	0.002
Number of severe hypoglycemic episodes since diagnosis (Mean ± SD)	0.17 ± 0.75	0.35 ± 1.13	0.13 ± 0.65	0.085
**Treatment in the past year, n (%)**				0.034
Hypoglycemic drugs alone	241 (48.8)	31 (36.0)	210 (51.5)	
Iinsulin alone	51 (10.3)	11 (12.8)	40 (9.8)	
Insulin with hypoglycemic drugs	189 (38.3)	43 (50.0)	146 (35.8)	
Other (diet, exercise, etc. )	13 (2.6)	1 (1.2)	12 (2.9)	
Insulin or insulin secretagogues, n (%)	366 (74.1)	75 (87.2)	291 (71.3)	0.002
Duration of insulin use (Mean ± SD, years)	3.82 ± 5.72	6.32 ± 6.76	3.30 ± 5.34	< 0.001
Duration of diabetes (Mean ± SD, years)	10.93 ± 7.44	12.22 ± 6.87	10.66 ± 7.54	0.029
Family history of diabetes, n (%)	260 (52.6)	48 (55.8)	212 (52.0)	0.515
**Number of hypoglycemic drugs used in the past year**				
(Mean ± SD)	1.68 ± 0.98	1.69 ± 1.02	1.68 ± 0.98	0.953
Biguanides, n (%)	367 (74.3)	58 (67.4)	309 (75.7)	0.110
Sulfonylureas, n (%)	103 (20.9)	15 (17.4)	88 (21.6)	0.392
Glinides, n (%)	63 (12.8)	15 (17.4)	48 (11.8)	0.151
Dipeptidyl peptidase-4 inhibitors, n (%)	46 (9.3)	12 (14.0)	34 (8.3)	0.103
Sodium-glucose cotransporter 2 inhibitors, n (%)	79 (16.0)	14 (16.3)	65 (15.9)	0.936
Alpha-glucosidase inhibitors, n (%)	136 (27.5)	28 (32.6)	108 (26.5)	0.251
Glucagon-like peptide-1 receptor agonists, n (%)	23 (4.7)	1 (1.2)	22 (5.4)	0.158
Thiazolidinediones, n (%)	13 (2.6)	2 (2.3)	11 (2.7)	1.000
Number of diabetes complications (Mean ± SD)	1.49 ± 1.30	1.86 ± 1.37	1.41 ± 1.27	0.005
Diabetic nephropathy, n (%)	132 (26.7)	29 (33.7)	103 (25.2)	0.106
Diabetic retinopathy, n (%)	132 (26.7)	31 (36.0)	101 (24.8)	0.032
Diabetic peripheral neuropathy, n (%)	231 (46.8)	53 (61.6)	178 (43.6)	0.002
Diabetic foot, n (%)	18 (3.6)	1 (1.2)	17 (4.2)	0.301
Diabetic macroangiopathy	222 (44.9)	46 (53.5)	176 (43.1)	0.079
IAH, n (%)	65 (13.2)	17 (19.8)	48 (11.8)	0.046

IAH, impaired awareness of hypoglycemia; FoH, fear of hypoglycemia; BMI, body mass index; HbA1c, glycated hemoglobin; SD, standard deviation. The independent sample t-test, the Mann–Whitney U test, Pearson’s chi-squared test, the continuity corrected chi-squared test, and Fisher’s exact test were used to compare differences between groups. Hypoglycemia was defined as a plasma glucose level ≤ 3.9 mmol/L in patients with diabetes and severe hypoglycemia was defined as any hypoglycemic episode that led to seizures, loss of consciousness, and/or urgent medical attention. The number of hypoglycemic episodes was self-reported by patients on a questionnaire based on blood glucose self-monitoring diaries and their own memories.

### Fear of hypoglycemia in patients with T2D

FoH was present in 86 of 494 patients with T2D according to the FH-15 score, indicating a prevalence of 17.4%. Furthermore, the prevalence of FoH in 95 (19.2%) patients with hypoglycemic episodes in the past two weeks was 40.0%. For the 399 patients who did not have hypoglycemia events in the past two weeks, the prevalence of FoH was 12.0%. A total of 247 (50.0%) patients had hypoglycemia events in the past year, and their prevalence of FoH was 26.3%. For patients without hypoglycemia events in the past year, the prevalence of FoH was 8.5%. Fifteen (3.0%) patients experienced severe hypoglycemic episodes in the past year, and the prevalence of FoH in them was 46.7%. The prevalence of FoH in patients without severe hypoglycemic episodes in the past year was 16.5%. Forty-six (9.3%) patients in the study experienced severe hypoglycemia events in the past, and the prevalence of FoH was 26.1%. For patients without past severe hypoglycemia experiences, the prevalence of FoH was 16.5%. Table 3 shows the prevalence of hypoglycemia and FoH in patients with T2D.

**Table 3:** The prevalence of hypoglycemia and fear of hypoglycemia in patients with type 2 diabetes.

	Hypoglycemic episodes				Severe hypoglycemic episodes			
	n	Past two weeks	n	Past year	n	Past year	n	Since diagnosis
Patients who had hypoglycemic episodes, n (%)	494	95 (19.2)	494	247 (50.0)	494	15 (3.0)	494	46 (9.3)
Patients who had hypoglycemic episodes with FoH, n (%)	95	38 (40.0)	247	65 (26.3)	15	7 (46.7)	46	12 (26.1)
Patients who had hypoglycemic episodes without FoH, n (%)		57 (60.0)		182 (73.7)		8 (53.3)		34 (73.9)
Patients who had no hypoglycemic episodes with FoH, n (%)	399	48 (12.0)	247	21 (8.5)	479	79 (16.5)	448	74 (16.5)
Patients who had no hypoglycemic episodes and no FoH, n (%)		351 (88.0)		226 (91.5)		400 (83.5)		374 (83.5)

FoH, fear of hypoglycemia.

The average total score of the FH-15 was 23.22 ± 8.53, and the average scores for the three domains (fear, interference, and avoidance) were 11.12 ± 4.20, 7.38 ± 2.94, and 4.72 ± 2.05, respectively. The three items with the highest average FH-15 scores were: how often are you afraid of having hypoglycemia while alone, how often do you fear not recognizing the symptoms of hypoglycemia, and how often do you stop doing things you used to do for fear of having a hypoglycemic episode^27^. Table 4 summarizes the score of the FH-15.

**Table 4:** Fear of hypoglycemia in patients with type 2 diabetes.

FH-15 items	Total, Mean ± SD (n = 494)	FoH, Mean ± SD (n = 86)
Total score of FH-15	**23.22 ± 8.53**	**37.56 ± 5.41**
Fear dimension	**11.12 ± 4.20**	**17.85 ± 3.05**
Interference dimension	**7.38 ± 2.94**	**11.87 ± 2.51**
Avoidance dimension	**4.72 ± 2.05**	**7.84 ± 1.81**
Item 1: how often do you fear not recognizing the symptoms of hypoglycemia	1.70 ± 0.84	2.73 ± 0.74
Item 2: how often are you afraid of not knowing what to do in the event of hypoglycemia	1.61 ± 0.73	2.47 ± 0.73
Item 3: how often are you afraid of having hypoglycemia at work	1.54 ± 0.76	2.38 ± 0.86
Item 4: how often are you afraid of having hypoglycemia outside of a hospital/health care setting	1.57 ± 0.76	2.53 ± 0.81
Item 5: how often are you afraid of having hypoglycemia while alone	1.76 ± 0.85	2.91 ± 0.71
Item 6: how often do you avoid social situations (meetings, outings, etc.) due to fear of having a hypoglycemic episode	1.56 ± 0.80	2.60 ± 0.90
Item 7: how often do you stop doing things you used to do for fear of having a hypoglycemic episode	1.67 ± 0.82	2.76 ± 0.74
Item 8: how often do you have hypoglycemia that makes you unable to drive or use machinery	1.35 ± 0.63	2.05 ± 0.91
Item 9: how often you have hypoglycemia that makes you unable to work	1.40 ± 0.65	2.22 ± 0.82
Item 10: how often do you have hypoglycemia that interferes with your leisure activities	1.57 ± 0.73	2.57 ± 0.70
Item 11: how often do you have hypoglycemia that interferes with your family life	1.51 ± 0.69	2.43 ± 0.66
Item 12: how often do you have hypoglycemia that interferes with your social life	1.54 ± 0.73	2.60 ± 0.69
Item 13: how often do you worry about losing consciousness due to hypoglycemia	1.57 ± 0.80	2.71 ± 0.85
Item 14: how often are you afraid of falling asleep for fear of having hypoglycemia at night	1.38 ± 0.60	2.12 ± 0.71
Item 15: how often are you afraid of taking a trip/holiday for fear of experiencing hypoglycemia	1.49 ± 0.73	2.48 ± 0.76

FoH: fear of hypoglycemia; SD: standard deviation; FH-15: Fear of Hypoglycemia-15 Scale.

### Factors associated with FoH in patients with T2D

Table 5 shows the results of univariate and multivariate logistic regression analysis. Variables with a *P* value < 0.1 in the univariate model, containing sex (*P* = 0.036), education level (*P* = 0.024), job status (*P* = 0.076), monthly household income per capita (*P* = 0.067), annual self-funded medical expenses (*P* = 0.014), cohabitation status (*P* = 0.006), number of hypoglycemic episodes in the past 2 weeks (*P* < 0.001), number of hypoglycemic episodes in the past year (*P* < 0.001), number of severe hypoglycemic episodes in the past year (*P* = 0.009), number of severe hypoglycemic episodes since diagnosis (*P* = 0.037), treatment in the past year (*P* = 0.008), insulin or insulin secretagogues (*P* = 0.003), duration of insulin use (*P* < 0.001), duration of diabetes (*P* = 0.078), number of diabetes complications (*P* = 0.004), diabetic retinopathy (*P* = 0.033), diabetic peripheral neuropathy (*P* = 0.003), diabetic macroangiopathy (*P* = 0.081), and IAH (*P* = 0.049), were included in the multivariate logistic regression analysis.

**Table 5:** Univariable OR and multivariable OR of patients with fear of hypoglycemia.

Variable	Univariable OR (95% CI)	*P* value	Multivariable OR (95% CI)	*P* value
Age	1.01 (0.99–1.03)	0.270	–	–
**Sex**				
Male	Referent	–	–	–
Female	1.65 (1.03–2.63)	0.036	–	–
BMI	1.03 (0.96–1.10)	0.456	–	–
HbA1c	0.95 (0.85–1.08)	0.435	–	–
Systolic blood pressure	1.01 (1.00–1.03)	0.134	–	–
Diastolic blood pressure	0.99 (0.97–1.02)	0.487	–	–
**Marital status**				
Single	Referent	–	–	–
Married	0.52 (0.13–2.01)	0.345	–	–
Divorced	0.18 (0.02–2.00)	0.162	–	–
Widowed	1.19 (0.27–5.26)	0.823	–	–
**Residential location**				
Urban	Referent	–	–	–
Rural	1.64 (0.71–3.79)	0.246	–	–
**Educational level**				
Elementary school	Referent	–	–	–
Middle school	0.85 (0.42–1.74)	0.658	–	–
High school	0.39 (0.17–0.88)	0.024	–	–
Junior college	0.91 (0.41–1.99)	0.807	–	–
Undergraduate college	0.72 (0.33–1.58)	0.414	–	–
**Job status**				
Full-time	Referent	–	–	–
Part-time	2.10 (0.21–21.10)	0.530	–	–
Retired	1.41 (0.82–2.42)	0.219	–	–
Other (job-waiting, etc.)	2.44 (0.91–6.56)	0.076	–	–
**Monthly household income per capita**				
< 1000 CNY (< 158.5 USD)	Referent	–	–	–
1000–2999 CNY (158.5–475.4 USD)	0.56 (0.18–1.77)	0.324	–	–
3000–5000 CNY (475.5–792.5 USD)	0.37 (0.12–1.16)	0.088	–	–
> 5000 CNY (> 792.5 USD)	0.34 (0.11–1.08)	0.067	–	–
**Annual self-funded medical expenses**				
< 2000 CNY (< 317.0 USD)	Referent	–	–	–
2000–5000 CNY (317.0–792.5 USD)	1.96 (0.81–4.70)	0.134	–	–
> 5000 CNY (> 792.5 USD)	2.84 (1.23–6.54)	0.014	–	–
**Sources of medical expenses**				
Self-funded	Referent	–	–	–
Medical insurance	0.83 (0.17–4.00)	0.820	–	–
New rural cooperative medical system	0.95 (0.15–5.94)	0.958	–	–
**Cohabitation status**				
Living with others	Referent	–	Referent	–
Living alone	2.64 (1.32–5.26)	0.006	2.48 (1.20–5.14)	0.015
Hypoglycemia health education (Referent: no)	1.46 (0.88–2.41)	0.140	–	–
Number of hypoglycemic episodes in the past two weeks	1.40 (1.18–1.68)	< 0.001	–	–
Number of hypoglycemic episodes in the past year	1.08 (1.05–1.12)	< 0.001	1.06 (1.03–1.10)	< 0.001
Number of severe hypoglycemic episodes in the past year	2.79 (1.29–6.02)	0.009	2.61 (1.20–5.69)	0.016
Number of severe hypoglycemic episodes since diagnosis	1.32 (1.02–1.71)	0.037	–	–
**Treatment in the past year**				
Hypoglycemic drugs alone	Referent	–	–	–
Iinsulin alone	1.86 (0.87–4.01)	0.112	–	–
Insulin with hypoglycemic drugs	2.00 (1.20–3.32)	0.008	–	–
Other (diet, exercise, etc. )	0.56 (0.07–4.49)	0.589	–	–
Insulin or insulin secretagogue (Referent: other measures)	2.74 (1.41–5.35)	0.003	–	–
Duration of insulin use	1.08 (1.04–1.12)	< 0.001	1.06 (1.02–1.10)	0.006
Duration of diabetes	1.03 (1.00–1.06)	0.078	–	–
Family history of diabetes (Referent: no)	1.17 (0.73–1.86)	0.516	–	–
Number of hypoglycemic drugs used in the past year	1.01 (0.79–1.28)	0.951	–	–
Biguanides (Referent: no)	0.66 (0.40–1.10)	0.111	–	–
Sulfonylureas (Referent: no)	0.77 (0.42–1.41)	0.393	–	–
Glinides (Referent: no)	1.58 (0.84–2.98)	0.154	–	–
Dipeptidyl peptidase-4 inhibitors (Referent: no)	1.78 (0.88–3.61)	0.107	–	–
Sodium-glucose cotransporter 2 inhibitors (Referent: no)	1.03 (0.55–1.93)	0.936	–	–
Alpha-glucosidase inhibitors (Referent: no)	1.34 (0.81–2.21)	0.252	–	–
Glucagon-like peptide-1 receptor agonists (Referent: no)	0.21 (0.03–1.55)	0.125	–	–
Thiazolidinediones (Referent: no)	0.86 (0.19–3.95)	0.845	–	–
Number of diabetes complications	1.30 (1.09–1.54)	0.004	–	–
Diabetic nephropathy (Referent: no)	1.51 (0.91–2.48)	0.108	–	–
Diabetic retinopathy (Referent: no)	1.71 (1.05–2.81)	0.033	–	–
Diabetic peripheral neuropathy (Referent: no)	2.08 (1.29–3.34)	0.003	–	–
Diabetic foot (Referent: no)	0.27 (0.04–2.06)	0.207	–	–
Diabetic macroangiopathy (Referent: no)	1.52 (0.95–2.42)	0.081	–	–
IAH (Referent: no)	1.85 (1.00–3.40)	0.049	–	–

HbA1c, glycated hemoglobin; BMI, body mass index; CNY, China Yuan; USD, US dollars; IAH, impaired awareness of hypoglycemia; OR, odds ratio; CI, confidence interval. Hypoglycemia was defined as a plasma glucose level ≤ 3.9 mmol/L in patients with diabetes and severe hypoglycemia was defined as any hypoglycemic episode that led to seizures, loss of consciousness, and/or urgent medical attention. The number of hypoglycemic episodes was self-reported by patients on a questionnaire based on blood glucose self-monitoring diaries and their own memories.

In the multivariate model, living alone (OR 2.48; 95% CI 1.20–5.14; *P* = 0.015), number of hypoglycemic episodes in the past year (OR 1.06; 95% CI 1.03–1.10; *P* < 0.001), number of severe hypoglycemic episodes in the past year (OR 2.61; 95% CI 1.20–5.69; *P* = 0.016), and duration of insulin use (OR 1.06; 95% CI 1.02–1.10; *P* = 0.006) were associated with FoH in patients with T2D.

## Discussion

ThE study evaluated the prevalence and factors associated with FoH in hospitalized patients with T2D. The cutoff score of FoH is 30.5 in the Chinese version of the FH-15, and the average score of the FH-15 in our sample was 23.22 ± 8.53, indicating that the overall FoH in patients in this study was at a low level. The prevalence of FoH in this study was 17.4%, lower than 27.7% to 45.4% estimated by several previous studies^23,25,31^. These studies included insulin-treated patients with T2DM and patients with T1DM, and our study included patients receiving various glucose-lowering therapies. However, patients receiving insulin or insulin secretagogues have a high risk of hypoglycemia and may be more susceptible to FoH^4,23^. Therefore, the different glucose-lowering therapies may be the reason for the overall low level of FoH and the lower prevalence of FoH in this study than that in other studies. Moreover, our results revealed that living alone, number of hypoglycemic episodes in the past year, number of severe hypoglycemic episodes in the past year, and duration of insulin use were associated with FoH.

The three items with the highest average FH-15 scores were: how often are you afraid of having hypoglycemia while alone, how often do you fear not recognizing the symptoms of hypoglycemia, and how often do you stop doing things you used to do for fear of having a hypoglycemic episode^27^. These results may reveal the most feared scenarios associated with hypoglycemia in patients with T2D. Based on these findings, the ability of patients to correctly recognize and independently deal with hypoglycemia is of great significance for improving their FoH. Therefore, health care providers should provide effective health education to patients with T2D to make them understand the causes of hypoglycemia, symptoms of hypoglycemia, and emergency treatment of hypoglycemia to reduce the occurrence of FoH effectively.

We found multiple factors correlated with FoH in patients with T2D. First, living alone was a factor associated with FoH in the present study, which was similar to the findings of Sakane et al.^23^. A possible explanation is that during a hypoglycemic episode, particularly when patients require help from others, patients living alone cannot obtain help from the people around them in time, likely making them fear hypoglycemia. Moreover, living circumstances such as living alone may be conducive to developing mental health problems in patients, thereby promoting the occurrence of FoH. Previous studies have shown that living alone is associated with high anxiety symptoms^32^, whereas anxiety symptoms are significantly associated with FoH^33,34^. In addition, collectivism is a traditional Chinese culture that emphasizes the coliving of family members, so the influence of living alone on the mental health of Chinese patients may be stronger^35^. Thus, the impact of living alone on FoH in patients with T2D can be considered from two main aspects: limited objective support and increased mental problems.

It is worth noting that the item that both the total participants and those with FoH in this study scored the highest on the FH-15 was: how often are you afraid of having hypoglycemia while alone^27^, indicating that hypoglycemic episodes while being alone may be the most feared scenario for patients with T2D in this study. When patients suffer from hypoglycemia alone, without help from others, the aversive symptoms may delay the patients' emergency response to hypoglycemia, and the ability of the patients to deal with hypoglycemia may also be limited, which likely results in adverse outcomes. However, patients being alone are not necessarily all living alone^35^, but living alone can reflect the degree of being alone to a certain extent. A qualitative study found that living alone means being alone for some elderly individuals^36^. Hence, there may be an overlap between the state of living alone and being alone, and this relationship may vary among different people. Health care providers not only need to focus more on the social support of patients who live alone, such as objective support for coping with hypoglycemia and subjective support for psychological status, but also take effective measures, such as instructing patients on how to manage hypoglycemia on their own, improving the autonomous emergency response ability of all patients, as well as increasing their confidence in facing hypoglycemia independently. Furthermore, health care providers should also track their psychological status after a hypoglycemia event to prevent and mitigate FoH.

Second, number of hypoglycemic episodes in the past year and number of severe hypoglycemic episodes in the past year were associated with FoH in our study. Several previous studies have explored the relationship between hypoglycemic episodes and FoH. Sakane et al.^23^ reported that severe hypoglycemic episodes during the past year were significantly related to FoH in patients with T2D. Grammes et al.^22^ found that the high-FoH group had more mild hypoglycemic episodes than the low-FoH group. Marrett et al.^37^ found that FoH levels increased with the severity of hypoglycemia in patients with T2D. Our study showed that number of hypoglycemic episodes in the past two weeks and the past year and number of severe hypoglycemic episodes in the past year in patients with FoH were significantly greater than in those without FoH. Moreover, a previous focus group discussion showed that severe hypoglycemia and nonsevere hypoglycemia could all result in FoH, and severe hypoglycemia might lead to more fear^38^. Thus, hypoglycemic episodes are closely related to FoH. Aversive symptoms and potential adverse outcomes of hypoglycemia may be the direct cause. Notably, symptoms such as hunger, tremors, sweating, dizziness, and palpitations associated with hypoglycemia, as well as convulsions, coma, or death in severe cases, bring significant discomfort and are life-threatening to patients^39^. Also, hypoglycemia may lead to adverse outcomes such as cardiovascular and cerebrovascular diseases, cognitive dysfunction, and vision loss, which colossally threaten the health of patients^40^. Therefore, hypoglycemia not only seriously affects the patient's daily life but also increases the economic burden and reduces the quality of life of the patients^41^.

In addition, Snoek^42^ reported that patients with FoH were divided into four subgroups: low risk for severe hypoglycemia and low level of fear, low risk for severe hypoglycemia and high level of fear, high risk for severe hypoglycemia and low level of fear, and high risk for severe hypoglycemia and high level of fear. In our study, as shown in Table 3, some patients experienced hypoglycemia or severe hypoglycemia but did not have FoH. In contrast, some patients did not experience hypoglycemia or severe hypoglycemia but had FoH, which was in line with Snoek's opinion^42^. It is noteworthy that FoH in patients with a low risk of severe hypoglycemia and a high level of fear is a phobic response that is not proportional to one's own real hypoglycemia risk and needs to be treated differently. It may be related to any external atmosphere associated with hypoglycemia, such as witnessing someone else's severe hypoglycemic episode. In our study, of the 86 patients with FoH, 74 did have experienced severe hypoglycemia. From Snoek's point of view^42^, most of the patients with FoH in this study had a phobic response: a state of low risk of severe hypoglycemia and a high level of fear. However, only nine patients in our study with no previous experience of hypoglycemia had FoH, which means that most patients with FoH had a history of hypoglycemia in the past (regardless of the degree of hypoglycemia). Different groups may show different fear responses to different degrees of hypoglycemia. Overall, the relationship between hypoglycemic episodes and FoH is not entirely ideal, and interventions for FoH should be individualized. For patients with extremely high levels of FoH, professional psychologists should be arranged to conduct psychological counseling to alleviate the patient's fear and make them return to the average level of phobic response. For patients with experience of hypoglycemia or high risks of hypoglycemia, health care providers should strengthen diabetes health education, optimize the treatment plan, and provide psychological care to prevent and reduce the occurrence of hypoglycemia and FoH.

Third, our study also found that duration of insulin use was associated with FoH in patients with T2D, similar to some previous studies. The survey of Bradley et al.^43^ showed that patients with T2D treated with insulin had higher FoH levels than those treated with diet or exercise alone, and Wang et al.^44^ and Fisher et al.^45^ also reported that patients who used insulin and insulin secretagogue had higher FoH levels. Erol et al.^46^ found that patients receiving intensive insulin therapy had more hypoglycemia worry and fear than those receiving conventional insulin therapy. It may be because insulin use increases the risk of hypoglycemia in patients with diabetes^47^. The longer the insulin is used, the greater the susceptibility to hypoglycemic episodes. The aversive symptoms and adverse outcomes of hypoglycemia may lead to FoH. Furthermore, the use of insulin may directly aggravate the psychological burden of patients and give patients psychological hints of hypoglycemia. Consequently, health care providers should attach great importance to patients who receive insulin or insulin secretagogues, improve health education, conduct patient-centered treatment, and regularly assess the psychological status to decrease the risk of hypoglycemia and improve FoH in patients.

We also found some differences between this study and previous studies^21,23,25,31,48–50^. First, although our results showed that the duration of diabetes was significantly longer in the FoH group than in the non-FoH group, multivariate logistic regression analysis did not find a significant relationship between duration of diabetes and FoH. A longitudinal study by Anderbro et al.^51^ found that the association of FoH with diabetes duration was stable for most patients, supporting the findings of the multivariate analysis of this study. However, Shi et al.^48^ reported that a longer duration of diabetes was correlated with higher levels of FoH in patients with T2D. Khunti et al.^52^ found that as the duration of diabetes increased, the rate of hypoglycemia also increased, and multiple studies have shown that hypoglycemia is an essential factor associated with FoH^23,31,37^. Therefore, the association between duration of diabetes and FoH may be because a more prolonged duration of diabetes is associated with a higher risk of hypoglycemia. Second, Al Hayek et al.^49^ and Gjerlow et al.^50^ found that females had high risks for FoH among patients with T1D. Female patients were twice as likely to have fear-related diseases as male patients, resulting from the interaction of various factors, such as physiology, environment, and social culture^53^. However, in our study, the female sex was not associated with FoH. However, the proportion of female patients in the FoH group was significantly higher than that in the non-FoH group. Thus, the effect of sex on FoH may be affected by a variety of other factors, and further research is needed. Health care providers should attach great importance to the effect of sex on FoH. Third, Wang et al.^21^ found that diabetes health education was related to FoH in patients with T2D, but this relationship was not significant in our study. Appropriate health education can make patients have a correct understanding and attitude toward hypoglycemia, while improper health education can make patients have an inadequate understanding and wrong attitude toward hypoglycemia, and this may be the reason for the controversial relationship between health education and FoH. Consequently, it is necessary to explore the influence of the quality and degree of health education on FoH to find the most effective health education program. In addition, Anarte et al.^31^ found that age above 40 increased the risk for FoH in patients with T1D. Castellano‑Guerrero et al.^25^ found that age 46–65 was a risk factor for FoH in patients with T1D. Al Hayek et al.^49^ found that patients with T1D aged 16–18 had a higher level of FoH than those aged 13–15. Wang et al.^21^ reported that patients with T2D < 60 had a higher level of FoH than those ≥ 60 years old. Thus, the effect of age on FoH is complex, which may be because different ages have different responsibilities, obligations, and thoughts, and there are differences in the situation between different individuals. Our study showed no significant difference in age between the FoH and non-FoH groups. Our participants were all T2D from a tertiary hospital, and they were generally older (60.04 ± 11.71 years old), which may be why the age of the two groups was not significant. Further, Castellano‑Guerrero et al.^25^ and Anderbro et al.^51^ found that IAH was associated with FoH in patients with T1D. However, our results showed that the relationship between IAH and FoH was insignificant in patients with T2D. It is worth noting that the item "How often do you fear not recognizing the symptoms of hypoglycemia"^27^ in the FH-15 was one of the three items with the highest scores, indicating that the participants in this study had some concerns about failing to recognize the symptoms of hypoglycemia. The inability of patients to accurately perceive the onset of hypoglycemia will increase the risk of adverse outcomes, and this uncertainty may induce FoH. Hence, it is also necessary to pay attention to the level of hypoglycemia awareness in patients with T2D.

This study has some strengths that provide practical guidance for health care providers. First, living alone is associated with FoH in patients with T2D. Therefore, health care providers should identify patients who live alone and provide them with appropriate diabetes health education, psychological evaluation, and skill guidance, teaching them relevant knowledge and skills to respond to hypoglycemic episodes with a positive attitude. Meanwhile, health care providers should also increase the knowledge and skills related to diabetes of the patient's relatives and friends, leading them to participate more in the disease-related situations faced by the patients. Second, the number of hypoglycemic episodes in the past year and the number of severe hypoglycemic episodes in the past year are associated with FoH. On the one hand, the experience of hypoglycemic episodes can be regarded as a signal for FoH high-risk groups, helping health care providers identify FoH in patients and those susceptible to FoH. On the other hand, the results illustrate the importance of preventing hypoglycemia. The uncomfortable symptoms of hypoglycemia and its harm to the body may be the root causes of FoH. Therefore, health care providers should adopt optimal patient-centered care measures to reduce hypoglycemia events. Furthermore, patients with an extremely high level of fear but a low risk of hypoglycemia should be referred to a psychiatrist to alleviate the abnormal fear responses. Third, duration of insulin use is also associated with FoH. Therefore, health care providers should provide patient-centered treatment and care to reduce the side effects of insulin and insulin secretagogues and carry out psychological care to enhance patients' confidence and reduce their psychological burden. Finally, there were multiple factors associated with FoH, and the development of relevant intervention measures should balance individualization and comprehensiveness.

This study also has several limitations. First, we could not establish causal relationships between research variables because of the cross-sectional design. Second, the samples in this study were hospitalized patients, which may limit the generalizability of the findings to non-hospitalized patients. Oravec et al.^54^ found that hospitalized patients with diabetes had higher comorbidity scores and prevalence of depression compared with non-hospitalized patients. However, diabetes patients with comorbidities are more prone to hypoglycemia, and depressive symptoms are associated with FoH^55,56^. Thus, compared with non-hospitalized patients, hospitalized patients may have poorer physical and mental health, affecting their attitudes and responses to hypoglycemia. Third, the number of hypoglycemic episodes was based on the blood glucose diary and memory, which may have caused memory bias. Therefore, further studies should choose more representative populations and use more objective methods to reduce bias.

## Conclusion

The prevalence of FoH in hospitalized patients with T2D was high, which is an issue that urgently needs to be solved in China. Our results indicated that patients living alone, patients with more hypoglycemic episodes in the past year, and patients with a longer duration of insulin use were associated with FoH. These findings provide a theoretical basis for preventing, identifying, and improving FoH in patients with T2D.

## Data Availability

The data used to support the findings of this study are available from the corresponding author upon request.
